# Myelofibrosis Treatment Algorithm 2018

**DOI:** 10.1038/s41408-018-0109-0

**Published:** 2018-07-31

**Authors:** Ayalew Tefferi, Paola Guglielmelli, Animesh Pardanani, Alessandro M. Vannucchi

**Affiliations:** 1 Division of Hematology, Department of Internal Medicine, Mayo Clinic, Rochester, MN USA; 2 Department of Experimental and Clinical Medicine, CRIMM, Center Research and Innovation of Myeloproliferative Neoplasms, Azienda Ospedaliera Universitaria Careggi, University of Florence, Florence, Italy

## Abstract

Two novel prognostic systems for primary myelofibrosis (PMF) were recently unveiled: GIPSS (genetically inspired prognostic scoring system) and MIPSS70 (mutation-enhanced international prognostic scoring system for transplant-age patients). GIPSS is based exclusively on genetic markers: mutations and karyotype. MIPSS70 includes mutations and clinical risk factors. In its most recent adaptation, the prognostic value of MIPSS70 has been bolstered by the inclusion of a three-tiered cytogenetic risk stratification and use of hemoglobin thresholds that are adjusted for sex and severity (MIPSS70+ version 2.0). GIPSS features four, MIPSS70 three, and MIPSS70+ version 2.0 five risk categories. MIPSS70 is most useful in the absence of cytogenetic information. MIPSS70+ version 2.0 is more comprehensive than MIPSS70 and is the preferred model in the presence of cytogenetic information. Both MIPSS70 and MIPSS70+ version 2.0 require an online score calculator (http://www.mipss70score.it). GIPPS offers a lower complexity prognostic tool that reliably identifies candidates for allogeneic stem cell transplant (GIPSS high-risk disease) or long-term observation with little or no therapeutic intervention (GIPSS low-risk disease). Ultimately, we favor a step-wise prognostication approach that starts with GIPSS but also considers MIPSS70+ version 2.0 for confirming the most appropriate treatment approach for the individual patient.

## Introduction

Primary myelofibrosis (PMF) is currently classified with polycythemia vera (PV) and essential thrombocythemia (ET) under the broad World Health Organization (WHO) category of myeloproliferative neoplasms (MPN)^1^. PMF results from clonal expansion of myeloid cells and is characterized by the variable presence of the driver mutations *JAK2*, *CALR*, or *MPL*, other mutations such as *ASXL1*, *SRSF2*, and *U2AF1*, morphologically characteristic megakaryocyte proliferation that might or might not be accompanied by reactive bone marrow fibrosis, peripheral blood leukoerythroblastosis, anemia, marked hepatosplenomegaly, and constitutional symptoms^2–4^. Survival is shortened in PMF, estimated at a median of 6 years, but is significantly longer in patients younger than age 60 years, estimated at a median of 15 years^5^. In addition to premature death, quality of life is often impaired in PMF, mostly because of constitutional symptoms and cachexia. Unfortunately, current drug therapy in PMF, including the use of JAK2 inhibitors, lacks disease-modifying activity; cure is only possible with allogenic hematopoietic stem cell transplant (HCT), which is by far the first-line treatment of choice for high-risk disease^6,7^. Considering the substantial risk of treatment-related mortality and morbidity, a reliable system of prognostication is needed to help with treatment decisions and justify the risk of HCT^8^.

## Prognostication in myelofibrosis: from IPSS to DIPSS-plus

In the last decade, several prognostic models for PMF have been introduced and have enabled clinicians to determine the most appropriate therapy for the individual patient. The International Prognostic Scoring System (IPSS) was first published in 2009 for application at the time of diagnosis^9^. IPSS includes five clinically derived risk variables: age >65 years, hemoglobin <10 g/dl, leukocyte count >25 × 10^9^/l, circulating blasts ≥1% and constitutional symptoms; the presence of 0, 1, 2, and ≥3 of these adverse features defines the IPSS risk categories of low, intermediate-1, intermediate-2, and high, with corresponding median survivals of 11.3, 7.9, 4, and 2.3 years. In 2010, IPSS was upgraded to the dynamic IPSS (DIPSS) using the same five risk variables^10^. Unlike IPSS, DIPSS can be used at any time during the clinical course of the disease and not only at time of diagnosis. In addition, DIPSS assigns two, instead of one, adverse points for hemoglobin <10 g/dl. Low, intermediate-1, intermediate-2, and high-risk DIPSS corresponds to 0, 1–2, 3–4, and 5–6 adverse points, respectively, with corresponding median survivals of not reached, 14.2, 4, and 1.5 years. In 2011, cytogenetic information was incorporated into the general framework of DIPSS in order to develop a more comprehensive DIPSS-plus model^11^. The latter included the same five clinical variables used in IPSS/DIPSS, but in addition considered three more DIPSS-independent risk factors: unfavorable karyotype, defined as the presence of complex karyotype or sole or two abnormalities that included +8, −7/7q−, i(17q), inv(3), −5/5q−, 12p− or 11q23 abnormalities, red cell transfusion need and platelet count <100 × 10^9^/l. DIPSS-plus low, intermediate-1, intermediate-2, and high-risk categories correspond to the presence of 0, 1, 2–3, and ≥4 of the above-mentioned eight risk factors, with respective median survivals of 15.4, 6.5, 2.9, and 1.3 years. Most recently, these traditional prognostic systems were shown to perform reasonably well in post-PV and post-ET myelofibrosis^12^, although other investigators have not confirmed the particular observation^13,14^.

## DIPSS-plus independent-risk factors

The effort to improve upon DIPSS-plus has resulted in the description of number of DIPSS-plus-independent-risk factors in PMF, including the absence of type 1/like *CALR* mutations^2,15–17^, presence of high-risk mutations including *ASXL1*, *SRSF2*, *U2AF1*Q157, *EZH2*, and *IDH1/2* (refs. ^18,19^), very high-risk (VHR) karyotype, defined previously by the presence of monosomal karyotype or inv(3)/i(17q) abnormalities^20^, but most recently refined to include single or multiple abnormalities of −7, i(17q), inv(3)/3q21, 12p−/12p11.2, 11q−/11q23, or other autosomal trisomies not including +8/+9 (e.g., +21, +19)^21^, degree of bone marrow fibrosis^22–24^, monocytosis^25^, markedly elevated serum lactate dehydrogenase^26^, nullizygosity for *JAK2* 46/1 haplotype^27^, low *JAK2*V617F allele burden^28,29^and increased serum levels of IL-8, IL-2R, free light chain and hepcidin^30–32^.

Among the above-listed variables, we focused on driver mutational status, high-risk mutations and karyotype, in order to develop contemporary risk models that included both molecular and cytogenetic information. In terms of driver mutations, in a recent evaluation of 709 consecutive Mayo Clinic patients with PMF, 467 (66%) harbored *JAK2*, 112 (16%) *CALR* type 1/like, 24 (3.4%) *CALR* type 2/like, 38 (5.4%) *MPL* mutations, and 68 (10%) were triple-negative^2^. The study confirmed that survival was significantly longer with type 1/like *CALR*, compared to all other driver mutations, which were otherwise similar in their prognosis. The adverse survival effect of not carrying the type 1/like *CALR* mutation was independent of *ASXL1* or *SRSF2* mutations, as well as DIPSS-plus, while the presence of the particular mutation partially alleviated the detrimental effect of *ASXL1/SRSF2* mutations^2^. DIPSS-plus independent high-risk mutations for survival in PMF always included *ASXL1* and *SRSF2*, and variably *EZH2*, *IDH1/2*, and *U2AF1*Q157^18,19^. The new prognostic models described below also considered the recently revised three-tiered cytogenetic risk stratification that was based on 1002 Mayo Clinic patients with PMF: “very high-risk (VHR)” karyotype included single or multiple abnormalities of −7, i(17q), inv(3)/3q21, 12p−/12p11.2, 11q−/11q23, or other autosomal trisomies not including +8/9 (e.g., +21, +19); “favorable” karyotype included normal karyotype or sole abnormalities of 13q−, +9, 20q−, chromosome 1 translocation/duplication or sex chromosome abnormality including −Y; and “unfavorable” karyotype included all other abnormalities, with corresponding median survivals of 1.2, 2.9, and 4.4 years^21^. The particular cytogenetics risk model was prognostically independent of current prognostic systems, as well as driver and high-risk mutations, and was also effective in predicting leukemic transformation.

## MIPSS70

MIPSS70 (mutation-enhanced international prognostic scoring system for transplant-age patients) is the newest and most contemporary prognostic system for PMF and includes clinical risk variables, in addition to mutations (MIPSS70) and karyotype (MIPSS70+ and MIPSS70+ version 2.0)^33,34^. MIPSS70, MIPSS70+, and MIPSS70+ version 2.0 were developed in patients age 70 years or younger, in order to be directly relevant for transplant decision making. MIPSS70 features nine variables, including three genetic (absence of *CALR* type 1/like mutation; presence of high molecular risk mutations, specifically *ASXL1, SRSF2, EZH2, IDH1*, or *IDH2*; and presence of ≥2 high molecular risk mutations) and six clinical risk factors (hemoglobin <10 g/dl; leucocytes >25 × 10^9^/l; platelets < 100 × 10^9^/l; circulating blast ≥2%; bone marrow fibrosis grade ≥2; and constitutional symptoms). Subsequently, hazard ratio weighted score of “2” was assigned to leucocytes >25 × 10^9^/l, platelets <100 × 10^9^/l, and presence of ≥2 high molecular risk mutations and a weighted score of “1” for all the other risk variables; a total score of 0–1, 2–4, and ≥5 defined the three-tiered MIPSS70 low-, intermediate-, and high-risk categories. The corresponding median survivals (5-year survival rates), in two separate patient cohorts were “not reached” (96%), 6.3 years (67%) and 3.1 years (34%), for the Mayo Clinic cohort, and 27.7 years (95%), 7.1 years (70%), and 2.3 years (29%), for the Italian patient cohort. When MIPSS70 was applied to all ages in the Italian patient cohort, 5-year survival rates were 91% for low-risk, 56% for intermediate-risk, and 23% for high-risk disease.

## MIPSS70+

MIPSS70+ includes cytogenetic information, in addition to mutations and some of the clinical risk variables included in MIPSS70 (ref. ^33^). The seven inter-independent risk variables for MIPSS70+ include four genetic (absence of *CALR* type 1/like mutation; presence of high molecular risk mutations, specifically *ASXL1, SRSF2, EZH2, IDH1*, or *IDH2*; presence of ≥2 high molecular risk mutations; and “unfavorable” karyotype) and three clinical risk factors (hemoglobin <10 g/dl; circulating blast ≥2%; and constitutional symptoms). “Unfavorable” karyotype in the context of MIPSS70+ was defined as VHR or unfavorable karyotype, according to the revised cytogenetic risk stratification for PMF. Subsequently, hazard ratio weighted score of “3” was assigned to unfavorable karyotype, a score of “2” was assigned to absence of *CALR*-type 1/like mutation, and presence of ≥2 high molecular risk mutations and a score of “1” was assigned to presence of high molecular risk mutations, hemoglobin <10 g/dl, circulating blast ≥2%, and constitutional symptoms; a total score of 0–2, 3, 4–6, and ≥7 defined the four-tiered MIPSS70+ low, intermediate, high, and very high-risk categories. The corresponding median survivals (5-year survival rates) in two separate patient cohorts were 20 years (91%), 6.3 years (66%), 3.9 years (42%), and 1.7 years (7%), for the Mayo Clinic cohort, and “not reached” (100%), 24.2 years (90%), 10.4 years (76%) and 3.9 years (47%), for the Italian patient cohort. When MIPSS70+ was applied to all ages in the Mayo patient cohort, 5-year survival rates were 85% for low risk, 63% for intermediate-risk, 33% for high-risk, and 5% for very high-risk disease^33^.

## GIPSS

Most recently, we have developed a genetics only-based prognostic system for PMF, the genetically inspired prognostic scoring system (GIPSS), which is exclusively dependent on mutations and karyotype^35^. In a Mayo-University of Florence, Italy collaborative study of 641 patients with PMF, who were informative for both cytogenetic and mutation information, multivariable analysis restricted to genetic risk factors identified VHR karyotype (HR 3.1), unfavorable karyotype (HR 2.1), absence of type 1/like *CALR* mutation (HR 2.1), presence of *ASXL1* (HR 1.8), *SRSF2* (HR 2.4), and *U2AF1*Q157 (HR 2.4) mutations as inter-independent risk factors for survival; *EZH2* and *IDH1* and *IDH2* mutations were not significant in the particular multivariable analysis. HR-weighted risk scores were subsequently assigned to VHR karyotype (2 points) and one point each for unfavorable karyotype, absence of type 1/like *CALR* mutation and presence of *ASXL1*, *SRSF2* and *U2AF1*Q157 mutations. Accordingly, the four-tiered GIPSS risk categories included low (zero points), intermediate-1 (one point), intermediate-2 (two points), and high (≥3 points), with corresponding median survivals (5-year survival rate) of 26.4 years (94%), 8.0 years (73%), 4.2 years (40%), and 2 years (14%) years. GIPSS was shown to perform as well as MIPSS70+, using conventional statistical measures of predictive accuracy. The study also revealed significant alignment of risk distribution between GIPSS and MIPSS70+; a patient with GIPSS “high”-risk disease was most likely to also be in the MIPSS70+ “high” or “very high”-risk category whereas a patient with GIPSS “low”-risk disease was almost certain to be in the MIPSS70+ “low”-risk disease category. However, the corresponding MIPSS70+ risk allocation was not predictable for GIPSS intermediate-1- and intermediate-2-risk disease. GIPSS was also shown to predict leukemic transformation.

## MIPSS70+ version 2.0

Although MIPSS70+ included cytogenetic information, the model did not capitalize on the additional prognostic contribution from VHR karyotype^21,33^. Furthermore, since our original report of MIPSS70/MIPSS70+^33^, we have identified *U2AF1*Q157 as an additional HMR mutation^19^ and defined new sex- and severity-adjusted hemoglobin thresholds. Accordingly, we have since revised MIPSS70+ into MIPSS70+ version 2.0 (ref. ^34^). Multivariable analysis of MIPSS70/MIPSS70+ relevant risk variables, after the incorporation of the above-mentioned new information, identified VHR karyotype, unfavorable karyotype, ≥2 HMR mutations, presence of HMR mutation, absence of type 1/like *CALR* mutation, constitutional symptoms, anemia adjusted for both sex and severity, and circulating blasts ≥2% as independent risk factors for survival. HR-weighted risk points were subsequently allocated to VHR karyotype (4 points), unfavorable karyotype (3 points), ≥2 HMR mutations (3 points), presence of an HMR mutation (2 points), absence of type 1/like *CALR* mutation (2 points), presence of constitutional symptoms (2 points), severe anemia defined by hemoglobin levels of <8 g/dl in women and <9 g/dl in men (2 points), moderate anemia defined by hemoglobin levels of 8–9.9 g/dl in women and 9–10.9 g/dl in men (1 point) and ≥2% circulating blasts (1 point). Subsequently, the sum of risk points for individual patients were considered in developing a new five-tiered MIPSS70+ version 2.0: very high-risk ≥9 points; high-risk 5–8 points; intermediate-risk 3–4 points; low-risk 1–2 points; and very low-risk zero points;^34^ in patients age 70 years or younger, the corresponding median survivals (10-year survival rates) were 1.8 years (<5%), 4.1 years (13%), 7.7 years (37%), 16.4 years (56%), and “median not reached” (92%).

## Risk-adapted treatment algorithms

**Fig. 1:**
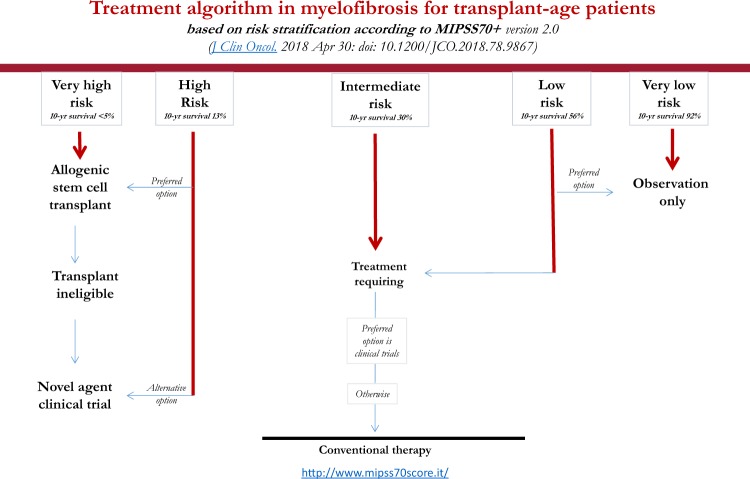
A contemporary treatment algorithm in myelofibrosis that employs MIPSS70+ version 2.0 (cytogenetic- and mutation-enhanced international prognostic scoring system for transplant-age patients)

**Fig. 2:**
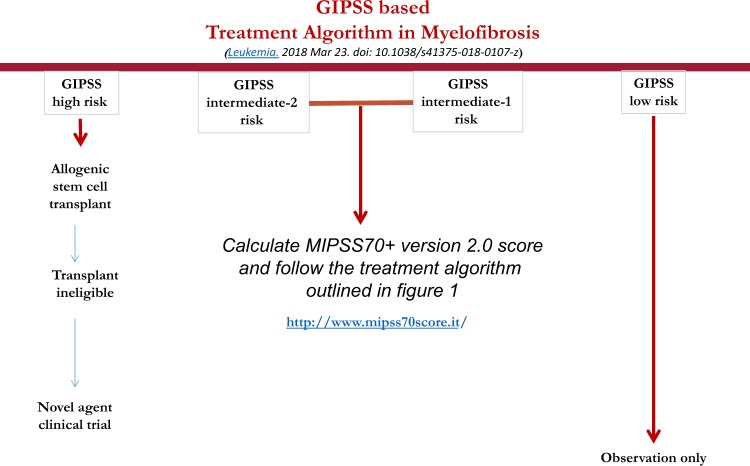
A contemporary treatment algorithm in myelofibrosis that employs GIPSS (genetically inspired prognostic scoring system)

**Fig. 3:**
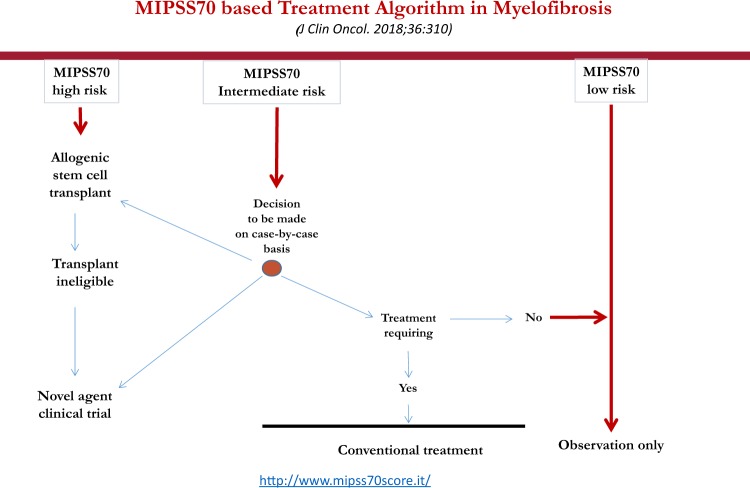
A contemporary treatment algorithm in myelofibrosis that employs MIPSS70 (mutation-enhanced international prognostic scoring system for transplant-age patients)

Table 1 summarizes the three most recent prognostic models in PMF. Figure 1 outlines an operational treatment algorithm that is based on the most recent adaptation of MIPSS70, which is MIPSS70+ version 2.0 (ref. ^34^). Fig. 2 proposes an alternative step-wise treatment algorithm that starts with the simpler-to-use GIPSS. In other words, because GIPSS high-risk disease always corresponds to MIPSS70+ very high- or high-risk disease^35^, additional prognostic information might not be necessary before recommending HCT for patients with GIPSS high-risk disease (Fig. 2). The same holds true for GIPSS low-risk disease, which also appears to always correspond to MIPSS70+ low-risk disease, and thus amenable to management with observation alone, especially considering the current lack of disease-modifying agents. Prognosis in patients with GIPSS intermediate-1- and intermediate-2-risk disease is too variable to forego a more comprehensive risk assessment using MIPSS70+ version 2.0 (Fig. 1) (http://www.mipss70score.it/). MIPSS70 (ref. ^33^) is best utilized in the absence of cytogenetic information but presence of molecular information; in this regard, we consider HCT as a reasonable and preferred treatment option for MIPSS70 high-risk disease and observation alone for MIPSS70 low-risk disease that is not requiring therapy (Fig. 3).

**Table 1:** Summary table for the three most recent prognostic models in primary myelofibrosis

	MIPSS70 (3-tiered)		MIPSS70+ *version 2.0* (5-tiered)		GIPSS (4-tiered)
	*Genetic variables*: One HMR mutation (1 point) ≥2 HMR mutations (2 points) Type 1/like CALR absent (1 point)	*Clinical variables*: Hemoglobin <10 g/dl (1 point) Leukocytes >25 × 10^9^/l (2 points) Platelets < 100 × 10^9^/l (2 points) Circulating blasts ≥2% (1 point) Constitutional symptoms (1 point) Bone marrow fibrosis grade ≥2 (1 point)	*Genetic variables:* VHR karyotype (4 points) Unfavorable karyotype (3 points) ≥2 HMR mutations (3 points) One HMR mutation (2 points) Type 1/like CALR absent (2 points)	*Clinical variables:* Severe anemia (2 points) Moderate anemia (1 point) Circulating blasts ≥2% (1 point) Constitutional symptoms (2 points)	Genetic variables: VHR karyotype (2 points) Unfavorable karyotype (1 point) Type 1/like CALR absent (1 point) ASXL1 mutation (1 point) SRSF2 mutation (1 point) U2AF1Q157 mutation (1 point)
Very low risk (median survival)			Zero points (not reached)		
Low risk (median survival)	0–1 points (not reached)		1–2 points (16.4 years)		Zero points (26.4 years)
Intermediate-1 risk (median survival)					One point (8 years)
Intermediate risk (median survival)	2–4 points (6.3 years)		3–4 points (7.7 years)		
Intermediate-2 risk (median survival)					Two points (4.2 years)
High risk (median survival)	≥5 points (3.1 years)		5–8 points (4.1 years)		≥3 points (2 years)
Very high risk (median survival)			≥9 points (1.8 years)		

MIPSS70: mutation-enhanced international prognostic system for transplant-age patients (age ≤70 years)^33^

MIPSS70+ version 2.0: mutation and karyotype enhanced international prognostic system (*J. Clin. Oncol.* 2018. doi: 10.1200/JCO.2018.78.9867). Survival quotes are for age ≤70 years

GIPSS: genetically inspired prognostic scoring system^19^. Survival quotes are for all age groups

HMR: high molecular risk mutations include *ASXL1*, *SRSF2*, *EZH2*, *IDH1*, *IDH2* and, in addition, for GIPSS and MIPSS70+ version 2.0, *U2AF1*Q157

VHR: very high-risk karyotype

Severe anemia: Hemoglobin <8 g/dl in women and <9 g/dl in men

Moderate anemia: Hemoglobin 8–9.9 in women and 9–10.9 in men

Intermediate-risk disease, according to either MIPSS70+ version 2.0 or MIPSS70, is managed based on the presence or absence of symptoms requiring therapy. In other words, observation alone is reasonable in the absence of treatment-requiring symptoms while clinical trial participation might be the best treatment approach in the presence of symptoms (Figs. 1–3). In patients deemed to be ineligible for HCT or clinical trial participation, symptom-directed conventional drug therapy, radiotherapy, or splenectomy is advised. These treatment options are palliative and unlikely to modify the natural history of the disease or prolong survival^36^. Nevertheless, anemia is best managed by the use of erythropoiesis-promoting drugs such as androgen preparations, danazol, thalidomide, and prednisone. Localized bone pain and symptomatic non-hepatosplenic extramedullary hematopoiesis responds well to involved-field radiotherapy. Ruxolitinib is effective in alleviating constitutional symptoms and marked splenomegaly^37^. Sooner or later, most patients become refractory to both hydroxyurea and ruxolitinib, and might require splenectomy. A recent study identified older age, leukocytosis, excess circulating blasts, and transfusion need as risk factors for inferior post-splenectomy survival^38^. For now, our approach to post-PV or post-ET is similar to that of PMF.

## Conclusion

Molecular signatures of tumors are finally being exploited in their diagnosis, prognostication, and treatment approach. In PMF, the WHO system has now formally included driver mutation screening in the diagnostic process. The current document illustrates the value of molecular information in the development and utility of genetic-based prognostic systems in PMF. There is also evidence that supports use of molecular information in the choice of specific treatment agents, although more studies are needed in that regard.
